# The Antitumor Effect of Xihuang Pill on Treg Cells Decreased in Tumor Microenvironment of 4T1 Breast Tumor-Bearing Mice by PI3K/AKT~AP-1 Signaling Pathway

**DOI:** 10.1155/2018/6714829

**Published:** 2018-04-23

**Authors:** Xin-ye Li, Liang Su, Yi-ming Jiang, Wen-bin Gao, Chun-wei Xu, Chang-qian Zeng, Jie Song, Yu Xu, Wen-cai Weng, Wen-bo Liang

**Affiliations:** ^1^Medical College of Dalian University, Dalian 116622, China; ^2^Xin Hua Affiliated Hospital of Dalian University, Dalian 116000, China; ^3^Department of Medical Oncology, The 3rd Affiliated Hospital of Shenzhen University, Shenzhen 518001, China; ^4^Department of Pathology, Affiliated Hospital of Academy of Military Medical Sciences, Beijing 100071, China

## Abstract

To study the antitumor effect of Xihuang pill (XHP) on the number of Treg cells in the tumor microenvironment of 4T1 breast tumor-bearing mice by PI3K/AKT/AP-1 pathway, a mouse model was established. Flow cytometry (FCM) and immunohistochemistry (IHC) were used to detect the number of Treg cells in the tumor microenvironment; terminal deoxynucleotidyl transferase dUTP nick end labeling (TUNEL) was used to detect the apoptosis of Treg cells in tumor microenvironment. Quantitative real-time PCR (RT-qPCR) was used to detect the mRNA expression of PI3K, AKT, and AP-1 in Treg cells in tumor microenvironment; immunofluorescence (IF) and Western Blot (WB) were used to detect the protein expression of PI3K, AKT, and AP-1 in Treg cells in tumor microenvironment. Compared with the naive control group, the tumor weight in XHP groups decreased significantly (*P* < 0.05); FCM and IHC results showed that the number of Treg cells in the tumor microenvironment decreased with the dose of XHP groups (*P* < 0.05); TUNEL staining showed that the number of Treg cells in tumor microenvironment increased with the dose of XHP groups (*P* < 0.05); RT-qPCR results showed that the mRNA expression of PI3K and AKT in Treg cells decreased with the dose of XHP groups, while RNA expression of AP-1 increased with the dose of XHP groups (*P* < 0.05); IF and WB results showed that the protein expression of PI3K and AKT in Treg cells decreased with the dose of XHP groups and the protein expression of AP-1 increased with the dose of XHP groups (*P* < 0.05). The results suggested that XHP decreased the number of Treg cells via inhibiting PI3K and AKT expression and upregulating AP-1 expression in Treg cells and then promoting the apoptosis of Treg cells. Thus, XHP could improve the immunosuppressive state of tumor microenvironment and reverse the immune escape to inhibit tumor growth.

## 1. Introduction

Cancer is one of the major killers of human health, and it is estimated that, by 2030, the global incidence of cancer is projected to reach 26.4 million, of which about 17 million people died from cancer [1]. Nowadays, breast cancer has become one of the most common malignancies in women, and about a half million people die each year from the disease (IARC Globocan, 2008) [2]. The modern medical therapies of breast cancer are endocrine therapy [3], targeted therapy [4], radiation therapy [5], and so on. Although the effect of modern medical treatment of tumor is remarkable, its adverse reaction is great; the body's immune system damage is serious. So to seek effective treatment of low adverse reactions and adjuvant therapy is very necessary.

Traditional Chinese medicine has a significant effect in adjuvant therapy for tumor, such as to improve clinical symptoms and prolong the survival of patients and regulate the immune function [6–8]. XHP was recorded in Wai Ke Quan Sheng Ji by Wang Hongxu during the Qing dynasty; it consisted of bezoar, musk, frankincense, and myrrh, commonly used in the treatment of cancer and adjuvant therapy [9]; XHP alone or its combination with Western medicine in the treatment of malignant tumors such as breast cancer, ovarian cancer, esophageal cancer, and other cancers has significant antitumor effect [10]. However, there is a little research on the mechanism of antitumor effect of XHP on breast cancer.

Tumor microenvironment is a complex network system composed of tumor cells, immune cells, extracellular matrix, and interstitial tissue. The metastasis, invasion, and development of tumor are related to tumor microenvironment [11, 12]. Treg cells are a subset of T lymphocytes with immunosuppressive function, which can inhibit the body's antitumor immunity and promote the immune escape from cancer cells in order to proliferate [13–15]. The increase of Treg cells can promote the formation of tumor microenvironment, and the tumor microenvironment can also induce the generation of Treg cells [16–18]. Studies have shown that an increase in the number of Treg cells in the cancer microenvironment is negatively correlated with the patient's survival [19–21]. Therefore, reducing the number of Treg cells will be an effective way in immunotherapy for tumors.

The phosphoinositide 3-kinase (PI3K)/AKT signaling pathway is an important intracellular cell proliferation pathway; activation of the PI3K/AKT signaling pathway can not only promote tumor cell proliferation [22, 23], but also play an important role in T cell proliferation [24, 25]. PI3K inhibitors can reduce the incidence of T cell-dependent pneumonia [26]; PI3K inhibitors and AKT inhibitors can inhibit the proliferation of Treg cells in human peripheral blood, thereby reducing the number of Treg cells [24]. The activation of AKT in T cells can exert antiapoptosis and promote tumor formation [27]. The expression of AKT inhibits the death of T cell hybridomas induced by the immediate early gene transiently Nur77 [28]. The downstream protein of the signaling pathway of PI3K/AKT is AP-1 [29], that is, a transcription factor [30]; it not only is associated with the incidence of breast cancer [31] but also participates in cell proliferation activities [32]. AP-1 plays an important role in TCR signaling pathway, lymphokine production, and lymphokine receptor signaling [33]. XHP was found to regulate the PI3K/AKT/AP-1 signaling pathway in T cell receptor signaling pathway of tumor microenvironment by mRNA high-throughput screening from our group. However, there is no report about the effect of XHP on PI3K/AKT/AP-1 pathway in Treg cells of tumor microenvironment. Therefore, the mechanism of XHP's antitumor effect was studied through focusing on the PI3K/AKT/AP-1 pathway which had impact on the number of Treg cells in the tumor microenvironment of tumor-bearing mice.

## 2. Materials and Methods

### 2.1. Animals, Cell Line, and Drug

Adult SPF grade BALB/c mice (female: aged 4–6 weeks, body weight 18–22 g) were purchased from the Laboratory Animal Center of Dalian Medical University (Dalian, Liaoning, China, License Number: SCKK (Liao) 2013-0003). The 4T1 mouse breast cancer cells were purchased from the Chinese Academy of Sciences cell bank. Xihuang pill was purchased from Tong Ren Tang Technologies Co. Ltd. (Beijing, China).

### 2.2. Model

The 4T1 mouse breast cancer cell lines were cultured in RPMI-1640 supplemented with 15% fetal bovine serum (FBS) and incubated at 37°C in 5% CO_2_. The cells were digested with Trypsin. The concentration of the cells was adjusted to 1 × 10^6^/ml with Phosphate Buffer Solution (PBS). Breast cancer models were established in the BALB/c mice divided into 4 groups (*n* = 10): three Xihuang pill groups and one model naive group; 0.2 mL (stated above) cells were inoculated into the right armpits of BABL/c mice. XHP groups were administrated with 0.39 g/kg, 0.78 g/kg, and 1.95 g/kg. Model naive group was given equal volume of distilled water. Since the second day after the model was established, the mice were treated with a daily dose of 2 ml/100 g twice a day consecutively for 14 days. At the fifteenth day, the tumor tissue was taken to carry out corresponding tests. The inhibition rates of tumor were calculated.

(1) Tumor  inhibition  rate=The  average  tumor  weight  in  the  navie  group−The  average  tumor  weight  in  the  administration  groupThe  average  tumor  weight  in  the  navie  group×100%.

### 2.3. Immunocytochemistry

Tumors were embedded with optimal cutting temperature compound (OCT), freezed with Drikold, and cut at a thickness of 7 *μ*m on cryostat microtome. Sections were fixed with 4% paraformaldehyde at room temperature for 20 min, followed by washing three times with PBS for 30 min. The membranes were permeabilized with 0.1% Triton X-100 on ice for 2 minutes, blocked with sheep serum at room temperature for 30 min, and incubated with Goat anti-mouse Foxp3 antibody (1 : 150, Novus, USA) at 4°C overnight, followed by washing three times with PBS. Then, sections were incubated with donkey anti-goat antibody (Alexa Fluor 488) (1 : 200, Jackson, USA) at room temperature (dark) for 1 h and washed the same way, counterstained with 4′,6-diamidino-2-phenylindole (DAPI), and washed three times. Antifluorescence quencher was added and covered with cover slips. The result was detected by the Olympus IX73 microscope (Tokyo, Japan).

### 2.4. Flow Cytometry

Single cell suspensions were prepared from 4T1 breast tumor and the concentration of them was adjusted to (2–10) × 10^8^ mL^−1^. Lymphocytes were isolated and washed using mouse tumor-infiltrating lymphocytes separation fluid (TBD Science, Tianjin, China) according to the manufacturer's instructions. The concentrations of 2 × 10^6^ mL^−1^ of lymphocytes were collected with PBS. Cells were incubated with Anti-Mouse CD16/CD32, antibody mixture (Anti-Mouse CD_4_^+^ FITC and Anti-Mouse CD_25_^+^ APC), Fixation/Permeabilization, and Anti-Mouse mouse/rat Foxp3 PE, in accordance with the manufacturer's instructions of eBioscience™ Mouse Regulatory T cell Staining Kit (Thermo Fisher Scientific, USA). After washing with permeabilization buffer, the cells were analyzed by flow cytometry FACSCalibur (BD, USA).

### 2.5. Treg Cells Were Sorted and Identified

The concentrations of the cells were adjusted to 1 × 10^8^ mL^−1^ with the RoboSep™ buffer (STEMCELL, Canada) and the next steps were both in accordance with the manufacturer's instructions of EasySep™ Mouse CD25 Regulatory T Cell Positive Selection Kit (STEMCELL, Canada). Last, the Treg cells were collected with PBS on ice.

Treg cells were dropped on slides and fixed with an amount of 4% paraformaldehyde for 5 min. Next, the cells were permeabilized with 0.1% Triton-X-100 for 30 min on ice. The cells were washed three times and were blocked with Immune Staining Blocking Buffer for 30 min. Then the cells were incubated with Goat anti-mouse Foxp3 (1 : 200, Novus, USA) overnight at 4°C, followed by three times washing. Next, they were incubated with Donkey anti-goat (Alexa Fluor 488) (1 : 200, Jackson, USA) at room temperature (dark) for 1 h and washed three times with PBS. And the cells were incubated with amount of DAPI at room temperature (dark) for 10 min. Last, antifluorescence quencher was added and covered with a cover slip; then the results were detected by Olympus IX73 microscope (Tokyo, Japan).

### 2.6. TUNEL Staining

Treg cells were dropped on slides and fixed with an amount of 4% paraformaldehyde for 1 h. Next, they were permeabilized with 0.1% Triton-X-100 for 2 min on ice. TUNEL staining was performed according to the manufacturer's instructions of In Situ Cell Death Detection Kit (Sigma, Germany) at room temperature (darker) for 1 h. Antifluorescence quencher was added and covered with cover slips; then the results were detected by Olympus IX73 microscope (Tokyo, Japan) and the number of apoptoses was determined per square millimeter.

### 2.7. Quantitative Real-Time PCR

Treg cells were harvested with 50 *μ*L PBS in the EP tube. The total RNA was extracted from the cells. Then, the PCR reverse transcription and the PCR reaction were applied both using the Power SYBR™ Green Cells-to-CT™ Kit (Invitrogen, USA) according to the manufacturer's instructions. The relative expression of each target gene was analyzed by 2^−ΔΔCt^ method using *β*-actin as the internal reference: ΔΔCt = (Ct target gene − Ct internal control) drug group − (Ct target gene − internal reference) naive group. The following are the forward and reverse primer sequences (AuGCT, Beijing, China): PI3K P110*α* (forward GATTTTGGGCACTTTTTGGA, reverse GCTGCCGAATTGCTAGGTAA), PI3K P85*α* (forward GAGATCGACAAACGCATGAA, reverse CACGTCTTCTCGTCATGGTG), AKT (forward GAGGATGCCAAGGAGATCAT, reverse CTGTGCCACTGGCTGAGTAG), C-JUN (forward ACGACCTTCTACGACGATGC, reverse GCCAGGTTCAAGGTCATGCT), and *β*-actin (forward CCTCTATGCCAACACAGTGC, reverse ACATCTGCTGGAAGGTGGAC).

### 2.8. Immunofluorescent Staining

Treg cells were fixed in 4% paraformaldehyde for 5 minutes, washed with PBS twice, and ruptured in Triton-X-100 at room temperature for 30 min. The cells were incubated for 30 min with immunofluorescent blocking solution. Next, they were incubated at 4°C overnight with different primary antibodies. After washing the primary antibodies, the cells were incubated with different secondary antibodies at room temperature for 1 hour. Then the cells were counterstained with 4′,6-diamidino-2-phenylindole (DAPI) and washed with PBS three times; the cells were added to antifluorescence quencher and covered with a cover slip, and the protein expression was detected by Olympus IX73 microscope (Tokyo, Japan). Image J was used for the quantitative analysis. The above antibodies were as follows: primary antibodies: Rabbit anti-mouse *β*-actin, Rabbit anti-mouse PI3K P110*α* (all 1 : 100, CST, USA), Rabbit anti-mouse C-JUN antibody, Rabbit anti-mouse AKT, and Rabbit anti-mouse PI3K P85*α* (all 1 : 100, Abcam, USA); secondary fluorescently labeled antibodies: Donkey anti-rabbit antibody (Alexa Fluor 488) and Donkey anti-rabbit antibody (Cy3) (all 1 : 200, Jackson, USA). Testing equipment was Olympus IX73 microscope (Tokyo, Japan).

### 2.9. Western Blotting Analysis

Treg cells proteins were extracted according to the Whole Cell Lysis Assay's instructions (KeyGEN BioTECH, Jiangsu, China). We took 50 *μ*g equal amount of protein for electrophoresis and transferred it onto polyvinylidene fluoride membranes. The membranes were blocked at room temperature for 1 h with Nonfat Dry Milk. The membranes were incubated overnight at 4°C with different primary antibodies in Nonfat Dry Milk (CST, USA) configured antibody dilution and incubated at room temperature for 1 h with secondary fluorescently labeled antibodies in the same antibody dilutions. The signal intensity was analyzed by Image J. The above antibodies were as follows: primary antibodies: Rabbit anti-mouse *β*-actin, Rabbit anti-mouse PI3K P110*α* (1 : 1000, CST, USA), Rabbit anti-mouse C-JUN antibody, Rabbit anti-mouse AKT, and Rabbit anti-mouse PI3K P85*α* (1 : 1000, Abcam, USA); secondary fluorescently labeled antibodies: Donkey anti-rabbit antibody (Alexa Fluor 790) and Donkey anti-rabbit (Alexa Fluor 680) (both 1 : 10000, Abcam, USA). The results were tested by Odyssey CLX S/N CLX-0926 (LICOR, USA).

### 2.10. Statistics

All experimental data were analyzed with SPSS17.0 statistical software and results are expressed as the mean ± SEM. The significance of differences between groups was evaluated using one-way analysis of variance (ANOVA) followed by a Least Significant Difference (LSD) test for comparison between groups. Differences were considered to be significant at *P* < 0.05.

## 3. Results

### 3.1. The Effects of XHP on Tumor Weight

**Figure 1:**
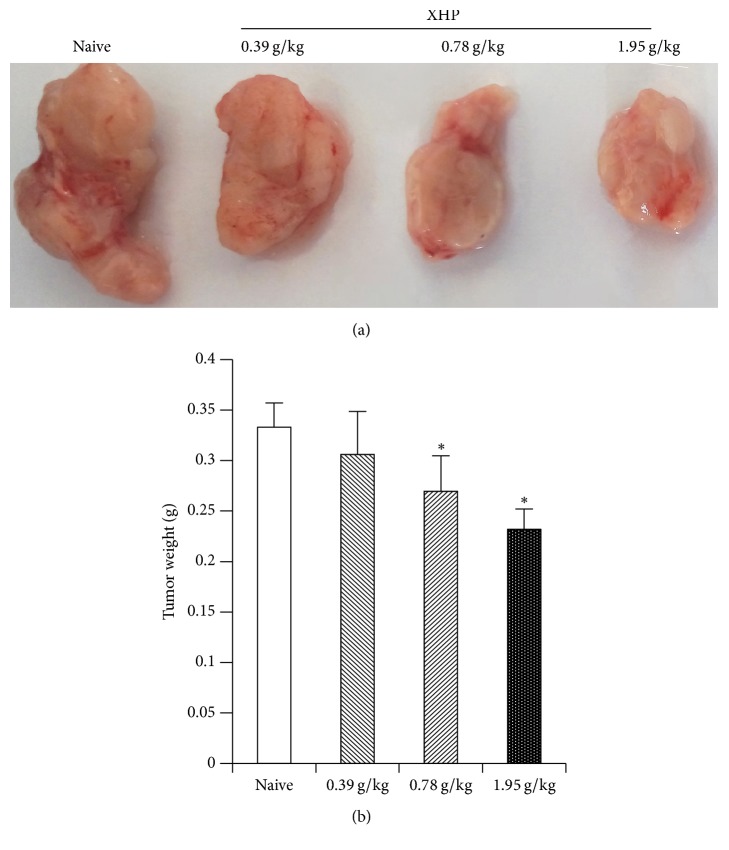
Effect of XHP on the growth of 4T1 mouse breast cancer. (a) Tumor size of different doses of XHP after treatment. (b) Different doses of XHP after treatment of tumor weight.

The tumor weight in XHP group administrated with the content of 0.78 g/kg and 1.95 g/kg was significantly reduced compared with the naive group (^*∗*^*P* < 0.05) (Figure 1).

### 3.2. The Effects of XHP on Number of Treg Cells in Tumor Microenvironment

**Figure 2:**
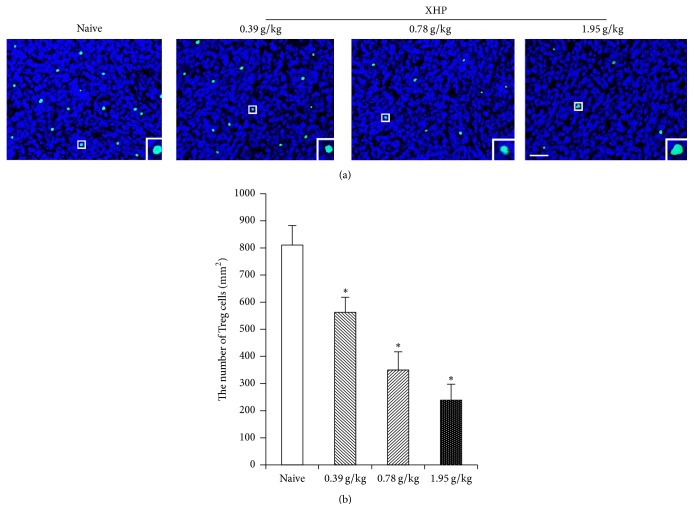
IHC detects the number of Treg cells by XHP on tumor microenvironment. (a) The results of IHC staining. (b) The number of Treg cells apoptoses per mm^2^ was quantified by cell count.

**Figure 3:**
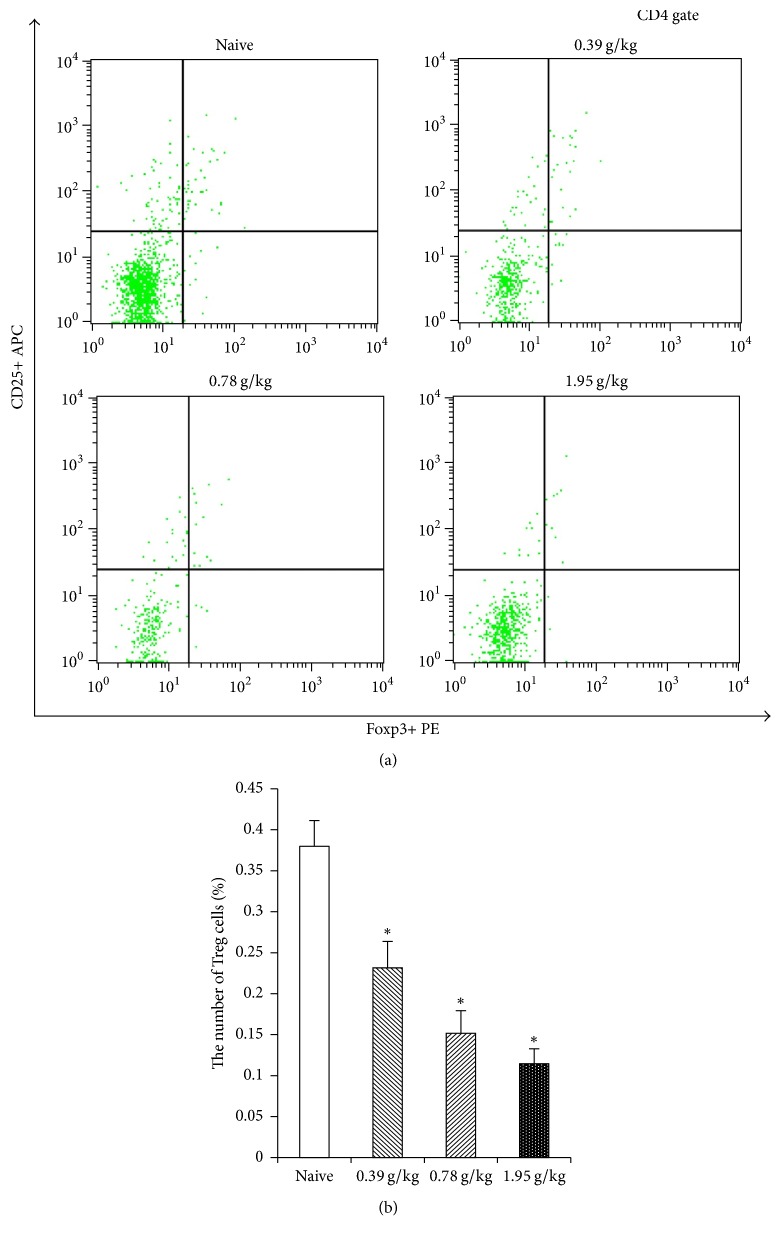
FCM detects the number of Treg cells by XHP on tumor microenvironment. (a) The results of FCM. Because Treg cells are a kind of lymphocyte that expressed CD4, CD25, and Foxp3 at the same time, in this experiment, we need to separate the single cell suspension of lymphoma for lymphocyte. After that, taking 2 × 10^6^ lymphocytes of each mouse in each group and CD4 gating for flow cytometry, the upper right quadrant is the Treg cells. (b) XHP groups on the number of Treg cells in the tumor microenvironment.

The number of Treg cells in tumor microenvironment of XHP groups which were administrated with the content of 0.39 g/kg, 0.78 g/kg, and 1.95 g/kg was significantly lower than the naive group (^*∗*^*P* < 0.05) (Figures 2 and 3).

### 3.3. The Identification of Treg Cells

**Figure 4:**
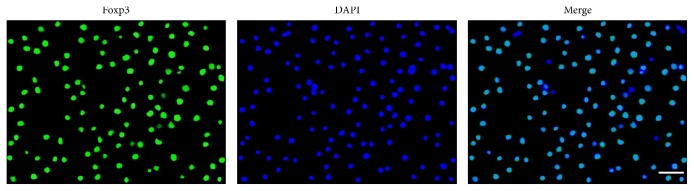
Blue: DAPI for the nucleus. Green: Foxp3+ cells. Blue-green: Merge. Scale: 20 *μ*m.

In order to ensure the follow-up of the experiment, we took the cells sorted by EasySep and incubated them with Foxp3 antibody to verify the purity of Treg cells in many preexperiments and formal experiments with the same experimental operating conditions; the results showed that the purity of isolated Treg cells was high. The positive rate of Treg cells was identified by transcription factor of Foxp3 that is specifically expressed on Treg cells. The average positive rate of Treg cells was 91.37  ± 4.01% (Foxp3+)/DAPI and was determined by Merge (Figure 4).

### 3.4. The Effects of XHP on Treg Cell Apoptosis in Tumor Microenvironment

**Figure 5:**
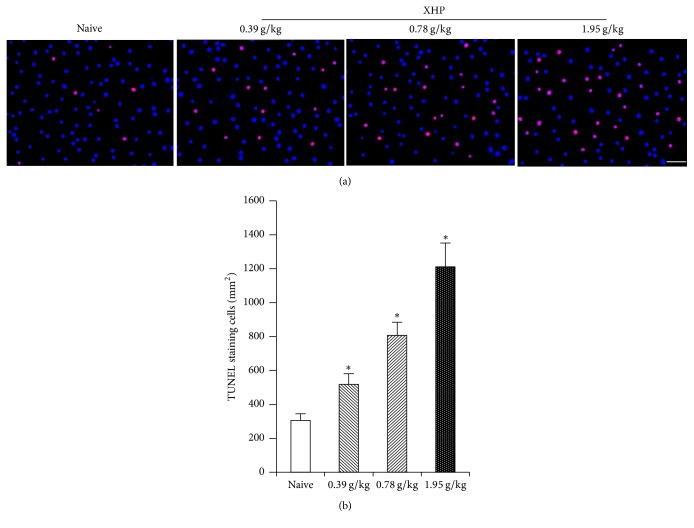
Treg cells apoptosis in tumor microenvironment. Scale: 20 *μ*m. (a) Treg cells apoptosis was detected by TUNEL staining. (b) The number of Treg cells apoptoses per mm^2^ was quantified by cell count.

The apoptosis rate of Treg cells in tumor tissue increased with the dose of XHP compared with the naive group (^*∗*^*P* < 0.05) (Figure 5).

### 3.5. Effects of XHP on mRNA Expression of P110*α*, P85*α*, AKT, and C-JUN in Treg Cells in Tumor Microenvironment

**Figure 6:**
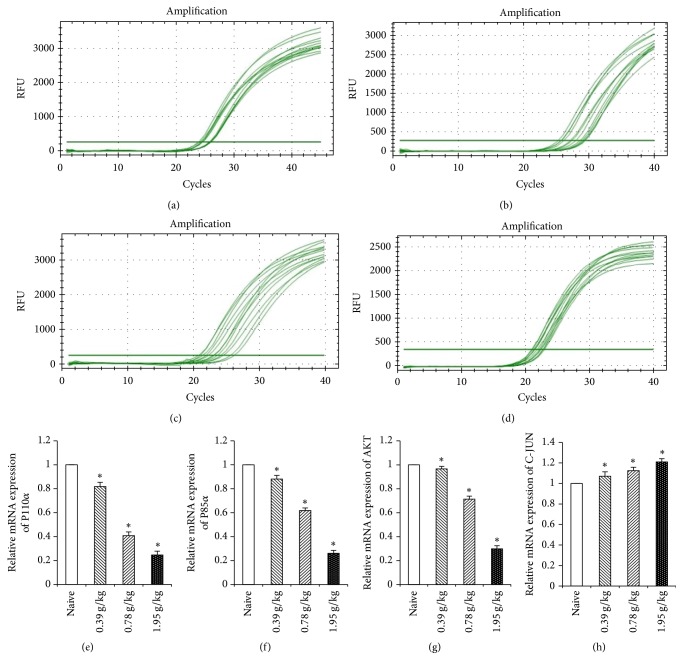
RT-q PCR amplification curve and gene expression relative quantitative analysis. (a), (b), (c), and (d): amplification curves of P110*α*, P85*α*, AKT, and C-JUN. (e), (f), (g), and (h): the relative mRNA expression of P110*α*, P85*α*, AKT, and C-JUN.

The mRNA expression of P110*α*, P85*α*, and AKT in Treg cells decreased with the increase of the dose of XHP in the tumor microenvironment and the mRNA expression of C-JUN increased with the dose of XHP compared with the naive group (^*∗*^*P* < 0.05) (Figure 6).

### 3.6. Effects of XHP on Proteins Expression of P110*α*, P85*α*, AKT, and C-JUN in Treg Cells of Tumor Microenvironment

**Figure 7:**
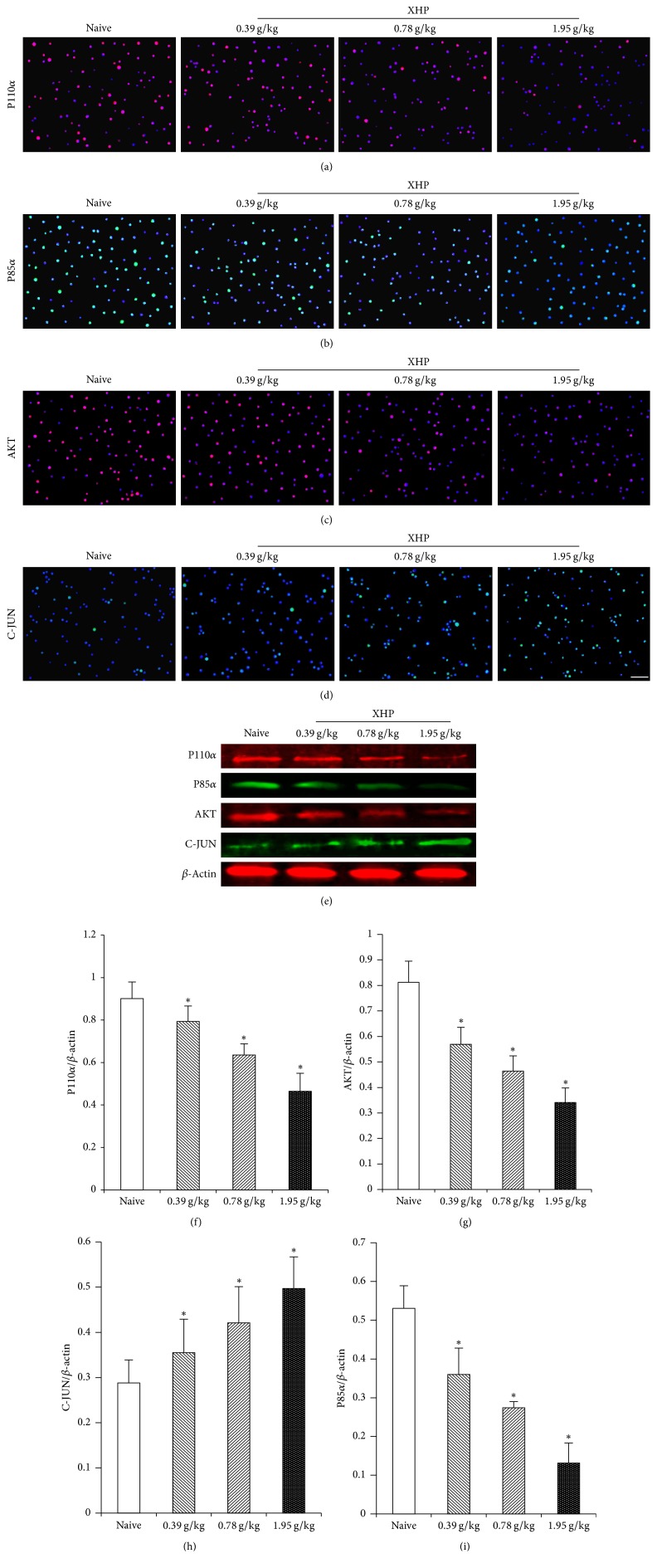
(a), (b), (c), and (d): the protein expression of P110*α*, P85*α*, AKT, and C-JUN on Treg cells in the tumor microenvironment by IF assay. (e): the protein expression of P110*α*, P85*α*, AKT, and C-JUN on Treg cells in the tumor microenvironment by WB. (f), (g), (h), and (i): the protein expression of P110*α*, AKT, C-JUN, and P85*α* in the Treg cells in the tumor microenvironment was expressed as the ratio of the gray level of each protein to the *β*-actin band.

The protein expression of P110*α*, P85*α*, and AKT in Treg cells decreased with the increase of the dose of XHP and the protein expression of C-JUN increased with the increase of dose of XHP compared with the naive control group (^*∗*^*P* < 0.05) (Figure 7).

## 4. Discussion

 XHP is the traditional famous option for the treatment of cancer. In vitro studies show that the water extract of XHP can induce apoptosis of Hs578T triple negative breast cancer cells through an intrinsic Bcl-2-independent pathway to play an antitumor effect [9]. In this experiment, XHP was found to reduce the tumor weight of 4T1 breast cancer in mice and the gradual increase of XHP dose can further reduce tumor weight in mice. The result shows that XHP can inhibit the growth of tumor by reducing the tumor weight.

The development of tumor is not only related to the tumor cells themselves, but also closely related to the environment in which they live (e.g., the tumor microenvironment) [34]. Tumor microenvironment is a complex “ecosystem” consisting of tumor cells, immune cells, stromal cells, and so on [12]. Treg cells were recruited into the tumor microenvironment with chemokines (such as CCL-28-CCR10) secreted by tumor cells and innate immune cells and thereby can proliferate in situ by using cytokines (such as IL-10 and TGF-*β*) in the tumor microenvironment, thus playing a role in the formation of tumor microenvironment [35, 36]. Treg cells can secrete cytotoxic T-lymphocyte-associated antigen 4 (CTLA-4), PD-1, and other immunosuppressive molecules and promote tumor escape by inhibiting the proliferation and activation of effector T cells [37–39]. Studies have shown that the number of Treg cells in the tumor microenvironment of invasive breast cancer and pancreatic cancer patients significantly increased; these Treg cells can inhibit the antitumor immune response, thus promoting the occurrence and development of tumors [40, 41]. Therefore, inhibiting the number and function of Treg cells may be an important target for regulating tumor microenvironment. Guo et al. [42] found that traditional Chinese medicine compound Feiyanning Decoction could decrease the number of Treg cells in mouse tumor tissue by flow cytometry. Another study had shown that XHP could improve the immunosuppressive status of tumor by reducing the number of MDSC cells in the tumor microenvironment. And the chloroform ethanol extract of XHP could increase the expression of IL-2 in Walker 256 breast cancer cells, reduce expression of IL-10, and improve the proportion of T lymphocytes, thus playing antitumor effect [43]. We tested the effects of XHP on the number of Treg cells in the tumor microenvironment of 4T1 breast cancer-bearing mice by FCM and IHC. The results showed that the number of Treg cells in XHP groups decreased, and the decrease of Treg cells was positively correlated with the dose of XHP. It is speculated that XHP may reduce the number of Treg cells in tumor microenvironment, thereby inhibiting tumor growth.

In order to elucidate the reasons for the decrease of Treg cells number, the apoptosis of Treg cells was tested after being treated by XHP. TUNEL staining was used to detect the apoptosis of Treg cells in the tumor microenvironment of 4T1 breast cancer-bearing mice. The results showed that the number of Treg cells apoptoses in XHP group increased and it was positively correlated with the dose of XHP. It is inferred that XHP may reduce the number of Treg cells by promoting the apoptosis of Treg cells in tumor microenvironment, thereby inhibiting tumor growth.

In order to clarify the mechanism of the apoptosis of Treg cells by XHP, the PI3K/AKT/AP-1 pathway in Treg cells was tested. PI3K is a complex that exists in the cytoplasm, consisting of regulatory subunit P85 and catalytic subunit P110 [22]; the activation of PI3K can promote a variety of cells proliferation and activation types [44]. In T cell-antigen presenting cell (APC) conjugates, phosphoinositide triphosphate (PIP3) accumulates in the T cell-APC synapse as well as the rest of the T cell plasma membrane. This suggests that there is abnormal regulation of PI3K activation during antigen presentation [45]. Silveira et al. [46] found that the increased activity of PI3K is associated with poor prognosis in primary T cell acute lymphoblastic leukemia (T-ALL). Serine/threonine kinase AKT is a common mediator of a variety of cell survival types [28]; AKT can directly regulate T cell size, metabolism, and function [47]. Studies have shown that reducing the AKT activity can inhibit T cell proliferation [25]. Evangelisti et al. [48] have found that triciribine (AKT inhibitor) alone or in combination with chemotherapy drugs can be used to treat T cell acute lymphoblastic leukemia. Rathmell et al. [47] found that the activated AKT could increase basal T cell metabolism and T cell metabolism and enhancement of T cell metabolism by AKT could promote the development of lymphoma. Simioni et al. [49] used flow cytometry, MTT, to detect MK-2206 (AKT inhibitor) on T cell acute lymphoblastic leukemia (T-ALL) cell lines; the results showed that MK-2206 can block the G0/G1 phase of leukemia cells, inducing apoptosis, thereby reducing the activity of T-ALL cell lines. AP-1 can regulate the proliferation of cells. Marusina et al. [50] found that the increased AP-1 can increase DAP10 activity in human T cells, thereby promoting the killing of tumor cells by NK cells and CD8 ^+^ T cells.

PI3K/AKT/AP-1 is an intracellular signaling pathway [29]. The activated P85 made p110 become recruited and activate on the cell membrane when intracellular receptor tyrosine kinase was activated, which could catalyze the formation of phosphoinositide triphosphate (PIP3) by phosphoinositide diphosphate (PIP2) on the inner surface of the membrane. PIP3, combined with the threonine T308 and serine S473 sites of AKT, transferred T308 and S473 to the cell membrane surface; thus the AKT-activating kinases PDK1 and PDK2 activated AKT, respectively [22, 51]. Activated AKT was inhibited by the combination of taurocholic acid (GCDC) and morphine deoxycholic acid (TCA), thereby increasing the activity of AP-1 [52]. Abu-Eid et al. [24, 29] found that PI3K inhibitor and AKT inhibitor of PI3K/AKT/AP-1 pathway can inhibit the proliferation of Treg cells, thereby enhancing the body's antitumor immune response and inhibiting tumor growth. The PI3K/AKT/AP-1 pathway was significantly changed when tumor-bearing mice interfered with XHP by mRNA high-throughput screening from our group. So, the Treg cells were isolated from the tumor microenvironment. The mRNA expression of PI3K/AKT/AP-1 in Treg cells was detected by RT-qPCR. The protein of PI3K/AKT/AP-1 expression in Treg cells was detected by IF and Western Blot. The results showed that the protein and mRNA expression of PI3K and AKT in Treg cells decreased and the protein and mRNA expression of AP-1 increased with XHP treatment.

## 5. Conclusion

The decrease of Treg cells in tumor microenvironment may be associated with Treg cells apoptosis induced by the expression of PI3K/AKT/AP-1 protein. It clarified that XHP could reduce the protein expression of PI3K and AKT in Treg cells in tumor microenvironment, increasing the protein expression of AP-1 to result in the apoptosis of Treg cells, thereby inhibiting the growth of tumor and clarifying the antitumor effect of XHP. The relationship of activation of PI3K, AKT, and AP-1 will be studied in our next experiments, which will provide better insights into the mechanism of XHP antitumor treatment and better references for the clinical antitumor treatment.
